# Predictors of Mortality in Patients With Necrotizing Fasciitis: A Literature Review and Multivariate Analysis

**DOI:** 10.1177/22925503211034830

**Published:** 2021-10-21

**Authors:** Lindsey Kjaldgaard, Nora Cristall, Justin P. Gawaziuk, Zeenib Kohja, Sarvesh Logsetty

**Affiliations:** 1 College of Medicine, Med II Research Program, Max Rady College of Medicine, Rady Faculty of Health Sciences, University of Manitoba, Winnipeg, Manitoba, Canada; 2 Manitoba Firefighters Burn Unit, Health Sciences Centre, Winnipeg, Manitoba, Canada; 3 College of Medicine, BSc (Med Research Program, Max Rady College of Medicine, Rady Faculty of Health Sciences, University of Manitoba, Winnipeg, Manitoba, Canada; 4 Department of Psychiatry, Max Rady College of Medicine, Rady Faculty of Health Sciences, University of Manitoba, Winnipeg, Manitoba, Canada; 5 Department of Surgery, Max Rady College of Medicine, Rady Faculty of Health Sciences, University of Manitoba, Winnipeg, Manitoba, Canada

**Keywords:** necrotizing fasciitis, mortality, epidemiology, critical care, multivariate analysis

## Abstract

**Background:**

Necrotizing fasciitis (NF) is a life-threatening infectious disease that can result in significant morbidity and mortality. Previously identified factors have not been verified in a large population. The objective of this study is to further examine the relationship of patient factors in NF mortality.

**Methods:**

This study is a retrospective review on patients ≥18 years old diagnosed with NF at the provincial referral centres from 2004 to 2016. The following data were examined: demographics, comorbidities, laboratory values, length of stay, and inhospital mortality.

**Results:**

Three hundred forty patients satisfied the inclusion criteria: 297 survived and were discharged, 43 died in hospital. In multivariate analysis, a prognostic model for NF mortality identified age >60 years, elevated creatinine, abnormal blood platelets, and group A β-hemolytic Streptococcus (GABS) infection.

**Conclusions:**

Multiple factors were associated with mortality in NF. The strongest univariate association with mortality was age >60 years. In addition, a history of hypertension and/or dyslipidemia, renal disease, and the presence of GABS contributed to a predictive model for inhospital NF mortality.

## Abstract (fr)

**Contexte:**

La fasciite nécrosante (FN) est une maladie infectieuse mettant la vie en danger et pouvant déboucher sur une morbidité et mortalité significatives. Les facteurs précédemment identifiés n’ont pas été vérifiés dans une large population. Cette étude avait pour objectif d’examiner plus en détail le rapport entre les facteurs liés au patient dans la mortalité de la FN.

**Méthodes:**

Il s’agit d’une étude rétrospective sur des patients âgés de ≥ 18 ans ayant eu un diagnostic de FN dans des centres de référence provinciaux de 2004 à 2016. Les données suivantes ont été examinées: Données démographiques, comorbidités, résultats des tests de laboratoire, durée du séjour à l’hôpital et mortalité à l’hôpital.

**Résultats:**

340 patients ont satisfait les critères d’inclusion: 297 patients ont survécu et ont reçu leur congé; 43 patients sont décédés à l’hôpital. Un modèle pronostique pour la mortalité dans la FN a identifié dans une analyse multifactorielle un âge > 60 ans, une élévation de la créatinine, des plaquettes sanguines anormales et une infection par des streptocoques β hémolytiques du groupe A (SGA).

**Conclusions:**

De nombreux facteurs sont associés à la mortalité dans la FN. L’association unifactorielle la plus forte avec la mortalité était un âge > 60 ans. De plus, des antécédents d’hypertension et/ou de dyslipidémie, une maladie rénale, et la présence de SGA ont contribué au modèle prédictif de la mortalité hospitalière liée à la FN.

## Background

Necrotizing fasciitis (NF) is an acute infection that can result in decreased quality of life, limb amputation, and death with mortality rates ranging from 5% to 72%.^1-4^ It would be of value to identify those patients at greatest risk of death who may benefit from a higher level of suspicion and more aggressive early intervention.

Necrotizing fasciitis spreads quickly throughout the fascia at up to rates of 2 to 3 cm/h, creating an urgency for diagnosis and management.^5^ It can go misdiagnosed 15% to 34% of the time,^6,7^ which can lead to delays in care and increase the risk of mortality.^8^ Once diagnosis is confirmed, wounds are managed in a similar fashion to a burn via skin grafting and/or free tissue transfer.^5,9^ Despite aggressive treatment, mortality remains high.^4^ With the incidence rates of NF in Manitoba previously reported as more than 5 times the Canadian rate, it is important to identify the factors that influence mortality in the Manitoban population.^4,9^

On reviewing the literature, 4 studies with >100 patients examined factors related to mortality in NF (Table 1). Jabbour et al conducted a retrospective review of 331 patients with surgically or histopathological confirmation of NF admitted to a surgical intensive care unit (ICU) in Qatar during 2000 to 2013. Mortality was defined as death in hospital (25.7%). This study examined diabetes, cardiovascular disease, renal failure, and older age. Using multivariate logistic regression, they found that age >60 years was a predictor of mortality (odds ratio: 1.06; 95% CI: 1.03-1.11).^1^ Due to Qatar being a high-income country of small size, shorter travel times may have resulted in quicker access to health care. The finding may differ from larger countries where transportation to definitive NF management can take several hours.

**Table 1.** Literature Review.

Authors	Jabbour et al (1)		Khamnuan et al (2)		Oud and Watkins (3)		Huang et al (4)	
Demographic								
n	331		1504		12172		472	
Mortality (%)	25.7		19.3		9.3		12.1	
Male (%)	74		56		54		67	
Age	50.8 ± 15.4		Unknown		Unknown		59.6	
Patient factors	Multivariate analysis (*P* value)	OR	Multivariate analysis (*P* value)	RR	Multivariate analysis (*P* value)	OR	Multivariate analysis (*P* value)	OR
Female gender	.928	0.952	.04	1.37				
Comorbidities, %								
Age > 60			<.001	1.39	.005 (age > 65)	1.491	.035	2.51
CV disease	.917	1.060	.003	1.64	<.0001	1.695		
Diabetes					.002	0.75		
Ethanol abuse					.30	1.165		
Liver disease			<.001	2.36	<.0001	3.204	<.001	9.74
Malignancy					<.0001	2.577		
Obese					<.0001	0.582		
Renal failure					<.0001	1.738		
Smoking history					.0017	0.603		
Laboratory values on admission								
aPTT > 60 seconds							.002	6.87
Creatinine (mg/dL) >1.6 mg/dL	.557	0.999					.019 (creatinine > 2.0 mg/dL)	2.69
Glucose (low)	.887	0.995	<.001	3.06				
Hemoglobin low (g/dL)	.416	1.088						
Sodium (mmol/L)	.442	0.965						
Vital signs on admission								
Systolic BP <90 mm Hg			<.001	2.05				
Pulse >130/min			<.001	2.26				

Abbreviations: aPTT, activated partial thromboplastin time; BP, blood pressure; CV, cardiovascular; OR, odds ratio; RR, relative rate.

Khamnuan et al examined 1504 patients with surgically confirmed NF from 3 general hospitals in Northern Thailand from 2009 to 2012. Mortality was defined as death in hospital or within 28 days of discharge. The mortality rate was 19.3%. Using a multivariate analysis, age >60 years, chronic heart disease, creatinine >1.6 mg/dL, female sex, liver disease, >130/min pulse on admission, and <90 mm Hg systolic blood pressure on admission were significant predictors of mortality. Although this study examined a large patient population, it was based in a middle-income country where access to care may be inconsistent. Therefore, the findings from this study may not be applicable to higher income countries.^10^

Oud and Watkins using administrative data studied 12 172 patients with NF admitted to Texas hospitals during 2001 to 2010.^11^ Patients were included based on the *International Classification of Disease* (*ICD*)*-9* codes for a NF episode. The mortality rate was lower than the previous 2 investigations at 9.3%. In multivariate regression, the factors significantly related to mortality were age >65 years, female sex, presence of congestive heart failure, diabetes, renal disease, and liver disease.^11^ The sample size is one of the largest to date on studies examining NF. However, the study has limitations as it did not have access to clinical data to examine the laboratory values and vitals on admission, microbiology, or outcomes related to amputations, grafts, and ICU admission. As well, Oud and Watkins did not include patients from privately funded hospitals or rural centres.

Huang et al studied 472 patients with NF admitted to a tertiary facility in Taiwan. Diagnosis of NF was confirmed by surgical observation. Mortality (12.1%) was defined as 30 days. Multivariate analysis showed significance for the following factors: cirrhosis, soft tissue air, *Aeromonas spp* infection, age >60 years, band polymorph nucleocytes more than 10%, activated partial thrombin time (aPTT) longer than 60 seconds, bacteremia, and serum creatinine >2.0 mg/dL.^12^ Similar to the other investigations, Taiwan has private health care and therefore access to care and resources for NF management may not be equal, which could affect mortality. All studies in this review occurred in settings that lacked universal health care leading to potential sample bias in attributing risk to various factors in NF mortality. Additionally, no study linked administrative data to clinical data to provide a comprehensive examination of risk factors for mortality.

## Methods

### Data Sources

Approval for this study was granted from the University of Manitoba Health Research Ethics Board. Patient data were collected from the NF Registry at the Health Sciences Centre in Winnipeg, Manitoba, Canada. The Registry includes information on patients with NF who are referred to one of the 2 provincial referral centres, capturing nearly all of provincial NF cases who survive to surgery. The registry is formed by identifying patients through health records who have been coded for diseases that may include NF. This included the following *ICD-9-Clinical Modification* codes and *ICD-10-Canadian Modification* (*ICD-10-CA*) codes for NF: ICD728.86, ICD-10-CA M72.6, Fournier gangrene (male, ICD-9-CM 608.83, ICD-10-CA N49.3; female, ICD-9-CM 616.89, ICD-10-CA N76.8) and myositis (M60). These *ICD* codes have been previously verified by our group.^9^ Each patient chart was reviewed to confirm documented evidence of necrotic fascia reported by the surgeon during surgical debridement and to collect the data to be evaluated. We did not include patients who died before surgery. Mortality was defined as “died in hospital” after surgical intervention.

### Patient Population

Patients ≥18 years who had a confirmed case of NF between January 1, 2004, and December 31, 2016, were included in the study. Two cohorts were established: survivors (n = 297) and deceased in hospital (n = 43).

### Data Collection

The following deidentified patient data were recorded:

1. Demographic: age, sex, regional health authority (determined by postal code of residence).
2. NF specific: percentage of total body surface area (%TBSA) affected, NF bacteriology.
3. Outcomes and resource utilization: number of procedures, amputations, flaps, grafts, other closures (defined as a primary closure which can include suturing), ICU admission, length of stay in ICU, length of stay in hospital, requirement of blood transfusion, number of units of packed red blood cells. ICU stay was defined as >12 hours in ICU.
4. Laboratory values and vital signs on admission: aPTT, albumin, creatine kinase, glucose, hemoglobin, international normalized ratio, myoglobin, platelets, pulse, respiratory rate, serum creatinine, temperature, urea, white blood cell count.
5. Pre-existing comorbidities: cardiovascular disease (ie, cardiomyopathy, congestive heart failure, ischemic heart disease, history of myocardial infarction, peripheral vascular disease), diabetes mellitus, history of smoking, hypertension and/or dyslipidemia, liver disease, obesity (body mass index >30 kg/m^2^), renal disease, substance misuse.

### Data Analysis

Data were analyzed using SPSS statistics version 25 (SPSS). Descriptive statistics were established and nominal associations were examined using Fisher exact test and Mann-Whitney *U* for nonparametric variables. Variables representing preexisting comorbidities, acute presentation, diagnostic values, and resource utilization were each examined separately as a predictor of mortality in univariate logistic regression with a *P* ≤.05 (2-tailed) considered significant. Missing data were excluded from analyses via pairwise deletion. This was followed by a series of multivariate logistic models with consideration in selecting factors with minimal missing values and limited multicollinearity. Critical values were established for serum creatinine and platelet ranges, and the values for these 2 factors were grouped into dichotomous values of normal and abnormal. Normal range for creatinine was considered 70 to 120 μmol/L (male) and 50 to 90 μmol/L (female), with 120 (male) and 90 (female) being the critical values.^13^ Normal range for platelets was considered 130 to 400 × 10^9^/L, with >400 × 10^9^/L or <130 × 10^9^/L being identified as critical values.^13^ Model fit was compared using the deviance between −2 log likelihood at a critical value of *P* ≤.05. Due to the large impact of age >60 years, model fit was compared both including and not including age >60 years in the model.

## Results

Demographic information on patients with NF is presented in Table 2.

**Table 2.** Demographic Characteristics Among Hospitalizations With NF.^a,b^

Age, years	50.4 (18.2-90.1)
Male	176 (51.8%)
%TBSA	5.2 (0.25-35.0)
Injury type	
Pain-redness	143 (43.1%)
Trauma: blunt	61 (18.3%)
Trauma: penetrating	41 (12.3%)
Preexisting skin ulcer	28 (8.4%)
Postoperative: other	16 (4.8%)
Postoperative: C-section	14 (4.2%)
Burn/frostbite	7 (2.1%)
Hysterectomy	5 (1.5%)
Not specified	17 (5.1%)
Bacteriology	
GABS	94 (27.6%)
GABS and other bacterium	22 (6.5%)
GBBS or GCBS	14 (4.1%)
*Escherichia coli*	12 (3.5%)
*Staphylococcus aureus*	29 (8.5%)
Polymicrobial	8 (2.4%)
Other	36 (10.6%)
Unidentifiable or no growth	125 (36.8%)

Abbreviations: GABS/GBBS/GCBS, Group A/B/C β-hemolytic Streptococci; NF, necrotizing fasciitis; TBSA, total body surface area.

^a^ n = 340.

^b^ Data shown as n (%) or mean (range).

Mean age was 50.4 (standard deviation [SD]: 15.4) years, 51.8% were male. Mean %TBSA was 5.2% (SD: 5.2). The most common organism was group A β-hemolytic Streptococcus (GABS) in 94 (27.6%) patients.

Comparison of health care resource utilization between the survived and deceased cohorts are shown in Table 3.

**Table 3.** Comparison of Demographics and Health Care Resource Utilization Between Cohorts. ^a,b,c,d^

	Survivors (n = 297)	Deceased (n = 43)
Age, years	48.6 ± 14.7	63.9 ± 12.2^e^
Sex, male	149 (50.2%)	27 (62.8%)
%TBSA	5.1 ± 5.2	6.1 ± 5.2
LOS, days	38 ± 40	28 ± 42
LOS/%TBSA	17.4 ± 33.0	16.3 ± 48.9
ICU stay, days	7.7 ± 11.9	9.8 ± 7.4
Blood transfusion, units	8 ± 7	8 ± 5
Procedures	3 ± 1	2 ± 2
Outcomes		
Amputation	32 (11.7%)^f^	7 (16.3%)
Graft	175 (63.6%)^g^	9 (20.9%)^e^
Free tissue transfer	19 (6.9%)^g^	2 (4.7%)
Other closure	82 (30.1%)^h^	2 (4.9%)^e,i^

Abbreviations: ICU, intensive care unit; LOS, length of stay; SD, standard deviation; TBSA, total body surface area.

^a^ Data shown as mean ± SD or n (%) or mean.

^b^ n = 40.

^c^ n = 42.

^d^ n = 271.

^e^ Significant difference, *P* < .05.

^f^ n = 274.

^g^ n = 275.

^h^ n = 272.

^i^ n = 41.

Of the 340 patients in this study, 43 died in hospital (12.7%). The deceased cohort was significantly older (*P* <.05). Survivors had a significantly higher rate of wound closure via graft, free tissue transfer, or other closure compared to the deceased cohort (*P* < .05). The deceased cohort also had a higher rate of ICU admission, more days in ICU, and received more packed red blood cells (*P* <.05).

Univariate logistic regression, exp(B), on patient mortality risk factors is shown in Table 4. Age >60 years, hypertension and/or dyslipidemia, renal disease, ICU admission, requirement of a blood transfusion, presence of GABS, elevated urea and creatinine, and abnormal platelets were all significantly associated with mortality (*P* <.05).

**Table 4.** Univariate Analysis of Mortality Risk Factors in Patients With NF.^a^

Factor	Survivors	Deceased	OR (95% CI)	*P*
Age >60 years	66 (23.7%)	27 (62.8%)	5.42 (2.75-10.67)	.001
Hypertension and/or dyslipidemia	122 (43.9%)	30 (69.8%)	2.95 (1.48-5.90)	.002
Renal disease	31 (11.2%)	13 (30.2%)	3.45 (1.63-7.31)	.003
ICU admission (% yes)	116 (45.5%)^b^	32 (76.2%)^c^	3.83 (1.81-8.13)	.001
Blood transfusion (% yes)	109 (40.2%)^d^	27 (62.8%)	2.51 (1.29-4.87)	.008
GABS (% yes)	102 (40.8%)^e^	8 (21.1%)^f^	2.32 (1.10-4.87)	.01
High creatinine	65 (36.7%)	18 (69.2%)	3.90 (1.50-9.40)	.007
Platelets, abnormal	58 (30.9%)	15 (6.0%)	3.40 (1.40-7.90)	.02

Abbreviations: ICU, intensive care unit; GABS, group A β-hemolytic *Streptococci* spp; NF, necrotizing fasciitis; OR, odds ratio; SD, standard deviation.

^a^ Data shown as n (%) or mean ± SD.

^b^ n = 255.

^c^ n = 42.

^d^ n = 271.

^e^ n = 250.

^f^ n = 38; creatinine n = 203, platelets n = 213.

A series of multivariate regressions with block entry were performed. Comparing critical values of χ^2^ difference in −2 log likelihood found that creatinine, blood platelets, the presence of GABS, and age >60 years provided the best model fit (Table 5). This was vastly improved in a final model employing a binary variable for creatinine (normal and high) and platelets (normal and abnormal). This final model provided 90% prediction in classification. In order of impact, high creatinine, presence of GABS, abnormal platelets, and age >60 years contributed to the final predictive model. The Nagelkerke *R* ^2^ indicates a 0.32 improvement in model fit with the predictors.

**Table 5.** Multivariate Logistic Regression Analysis of Mortality Risk Factors in Patients With NF.

Factor	*B*	Wald χ^2^ test	OR (95% CI)	*P*
High creatinine	1.78	9.41	7.94 (1.46-20.01)	.002
GABS (yes)	1.83	6.47	6.24 (1.52-25.5)	.011
Platelets, abnormal	1.76	9.41	5.80 (1.89-17.83)	.002
Age >60 years	1.46	7.27	4.29 (1.49-12.35)	.007

Abbreviations: GABS, group A β-hemolytic *Streptococci* spp; OR, odds ratio.

## Discussion

This study is the first retrospective review to examine the factors associated with mortality in NF in a Western country with universal access to health care. This is in contrast to previous studies done in Thailand and Qatar.^1,10^ Compared to other studies that have examined outcomes in patients with NF, this study is one of the largest.^14-16^

Consistent with other investigations, GABS was the most common organism isolated.^1,10,17^ However, 36.5% of patients did not have an identifiable organism on culture. This may be because almost half of the patients in the study were transported from various locations in the province at one of 2 provincial referral centres. With a province 1.5 times the area of California, travel time to the referral centres can take up to 12 hours. Many individuals received antibiotics prior to arrival, resulting in a negative tissue culture on admission.

Factors such as vitals on admission, location of infection, and sex were not found to be significant. Other comorbidities reported in previous studies as significant such as diabetes, smoking, malignancy, ethanol abuse, and liver disease^1,10,11^ were also not found to be significantly related to mortality in the current study. However, univariate analysis shows several factors were found significantly associated with mortality: including age >60 years, hypertension and/or dyslipidemia, renal disease, ICU admission, requirement of a blood transfusion, a causative organism of GABS, elevated urea and creatinine, and abnormal platelets. Age >60 years, low platelets, and elevated creatinine have been previously reported as risk factors for NF mortality.^1,10,11^ This study is unique in establishing GABS infection as well as the requirement of blood transfusions as risk factors in NF mortality.

In multivariate analysis, a prognostic model identified the following 4 risk factors for NF mortality: age >60 years, elevated creatinine, abnormal blood platelets, and GABS infection. Elevated creatinine has been shown to increase the risk of mortality in other disease processes. Studies have determined its importance in cardiac surgery and liver transplants regarding patient prognosis.^18,19^ Serum creatinine levels in a patient with NF may be elevated due to preexisting kidney failure or inadequate resuscitation.^20^ With the knowledge of its role in mortality for NF, creatinine levels in patients with NF require further investigation and may involve more aggressive management for patients with elevated creatinine such as earlier renal replacement therapy.

This is among the first reports in the literature of a significantly increased risk in mortality with abnormal levels of platelets. Low platelets have been previously reported as a predictor for mortality in NF.^1^ However, this study found that either high or low platelet count is significantly associated with increased risk of mortality in NF.^21^ The association of high platelets (thrombocytosis) as a risk factor for mortality has been noted in other critical illnesses.

Previous studies on smaller patient populations have found that *Clostridium* spp, *Vibrio* spp, and *Aeromonas* spp may increase the risk of mortality with NF.^2,12,22^ The current study is the first to determine GABS as a significant risk factor for NF mortality in a larger patient population. Using this knowledge, patients with GABS infections and multiple comorbidities can be more closely monitored due to increased risk of dying in hospital.

## Strengths

This is one of the first population-based retrospective reviews of NF mortality with universal health care: in a province, with single payer insurance and only 2 referral centres for all patients with NF. Another strength is that this study is based on a combination of clinical data and administrative data, bringing information from multiple sources. By including only cases with surgically confirmed NF, the current study results in a more homogeneous group. This varies from investigations that were more inclusive and also had more superficial necrotizing soft tissue infection (NSTI) cases in the study cohort.^2,22-24^ Finally, a strength to this study is that due to all of the patients in Manitoba with NF being referred to one of 2 tertiary centres covered by the same consulting group, all patients were managed with the same protocol. This lessens the likelihood of varied management practices, which could be a confounding factor in other studies.

## Limitations

The most significant limitation of this study is that some of the patients were identified and their data were collected retrospectively through chart review. Consequently, not all patients had all the variables of interest, limiting our ability to assess all factors. We are also unable to assess the variables that are not routinely tested for in the institutions involved in this study (ie, C-reactive protein, albumin). In addition, given the high odds ratio and sample size, there was also a likelihood of overfitting the model. Low-risk patients may have been underestimated and the relatively high odds ratios may indicate a possible overestimation for high-risk patients.^25^ A strength of our manuscript is that all patients with NF in the province are referred to one of the 2 sites in our study, resulting in the capture of nearly the entire population. However, because of the large geographical catchment area (approximately 251 000 square miles) and inclement winter weather, transport times can be prolonged. We did not have a reliable way of identifying the time from the start of the infection to definitive treatment to evaluate the possible effect of transport time on mortality.

## Future Directions

Future research examining NF mortality could involve building a prognostic scale that can be used by health care professionals. This has been attempted in the past by Golden et al, but involved a small sample size of less than 100 patients.^26^ A future prognostic scale could be made using the methods that were used in the past to create tools for NSTIs and toxic epidermal necrolysis.^27,28^

## Conclusions

This study presents an analysis of risk factors in one of the largest known cohorts of patients with NF using a combination of clinical and administrative data. Age >60 years, elevated creatinine, abnormal platelets, and GABS infection were shown to significantly increase the risk of mortality in patients with NF.
